# CoNet app: inference of biological association networks using Cytoscape

**DOI:** 10.12688/f1000research.9050.2

**Published:** 2016-10-14

**Authors:** Karoline Faust, Jeroen Raes

**Affiliations:** 1 Center for the Biology of Disease, VIB, Leuven, 3000, Belgium; 2 Microbiology Unit, Faculty of Sciences and Bioengineering Sciences, VUB, Brussel, 1050, Belgium; 3 Department of Microbiology and Immunology, REGA Institute, KU Leuven, 3000, Belgium

**Keywords:** network generation, network construction, network inference, association networks, microbial networks, CoNet, Cytoscape

## Abstract

Here we present the Cytoscape app version of our association network inference tool CoNet. Though CoNet was developed with microbial community data from sequencing experiments in mind, it is designed to be generic and can detect associations in any data set where biological entities (such as genes, metabolites or species) have been observed repeatedly. The CoNet app supports Cytoscape 2.x and 3.x and offers a variety of network inference approaches, which can also be combined. Here we briefly describe its main features and illustrate its use on microbial count data obtained by 16S rDNA sequencing of arctic soil samples. The CoNet app is available at: http://apps.cytoscape.org/apps/conet.

## Introduction

The analysis of species abundance patterns has a long tradition in ecology ( Connor & Simberloff, 1979; Diamond, 1975; Gotelli & McCabe, 2002). To the best of our knowledge, Jared Diamond was the first to infer an ecological relationship, namely competition, from mutual exclusion patterns in the distribution of tropical bird species ( Diamond, 1975). Since then, co-occurrence analysis, which looks for significant co-presence or mutual exclusion, has become a widely applied technique in ecology (e.g. ( Horner-Devine *et al.*, 2007)).

Co-occurrence analysis is an instance of network inference, which predicts relationships between objects from repeated measurements of objects' presence or abundance. Recent sequencing projects quantified the abundance of hundreds of microbial taxa by counting marker genes (usually 16S rDNA) sequenced in a large number of samples (e.g. ( Gilbert *et al.*, 2014; Human Microbiome Project Consortium, 2012)) These large sample numbers open the way to unraveling the complex relationships between microorganisms from their abundances across samples. CoNet was developed to carry out microbial network inference from sequencing data, but its generic design makes it applicable to any data set where objects have been observed repeatedly.

The construction and interpretation of microbial networks from sequencing data faces a number of challenges ( Faust & Raes, 2012). Since a different amount of DNA is sequenced in each sample, microbial marker gene counts have to be normalized to adjust for varying sequencing depth. This normalization in turn makes the count data compositional, which distorts correlation measures ( Friedman & Alm, 2012). In addition, an edge in a microbial network does not necessarily represent an ecological interaction such as mutualism or competition, since it may also be indirect, i.e. resulting from the response of two taxa to an environmental factor or another taxon. A recent evaluation has shown that the accuracy of ecological interaction inference from simulated sequencing data is low ( Weiss *et al.*, 2016). However, despite these limitations, network inference can give interesting insights into what shapes community structure, as we hope to demonstrate with our use case.

## Methods/Implementation

CoNet is implemented as a command line tool, which is wrapped by the CoNet app. The command line and Cytoscape 2.× app version are implemented in Java 1.6, whereas the Cytoscape 3.× app version requires Java 1.7.

### Implementation challenges and decisions

In general, the CoNet app is designed with minimum contact to Cytoscape, to ensure consistent behavior across different Cytoscape versions and to ease porting to future Cytoscape versions. The CoNet app is linked to Cytoscape only via its main menu and graph visualization classes. The Cytoscape-version-specific implementation of the graph visualization class is loaded via reflection at run time and is entirely separated from graph generation.

A major challenge for the implementation of the CoNet app is inclusion of the large number of options available in CoNet, which allows users to customize each network inference step, from data preprocessing via threshold setting, network construction and assessment of significance. This problem was solved by implementing a single user input handling class, which collects and checks user input from the various menus and submits it to CoNet once the GO button is pushed. This design allows to export and to read in user settings files, which make experiments carried out with the CoNet app more reproducible.

Another challenge is the command line support. Network inference from large data sets is not feasible within Cytoscape and CoNet is best run on command line for these cases. To facilitate this step for the inexperienced user, the current settings of the CoNet app can be exported as a command line call, by clicking the "Generate command line call" button. This call can then be executed on command line by including the CoNet jar file in the class path. Networks generated on command line can be loaded either via Cytoscape network import functions (if saved in gml format (( Himsolt)) or more conveniently via the CoNet app (if saved in the custom gdl format). The CoNet app's manual includes a step-by-step tutorial for command line usage.

The CoNet app also integrates the popular network inference R Bioconductor package minet ( Meyer *et al.*, 2008). We decided to integrate it loosely via Rserve, a Java-R bridge capable of transferring R objects to Java and *vice versa* ( http://rforge.net/Rserve/). Thus, advanced users can install and launch the Rserve server in R and configure the Rserve client settings (i.e. host and port) in CoNet app's configuration menu. The CoNet app's manual explains Rserve installation and usage.

Finally, we also implemented solutions for error and help display. The CoNet app displays help pages in html format, which allows the user to follow links within these pages. The CoNet app's pdf manual is compiled from the help pages using prince ( http://www.princexml.com/). Each menu is linked to its specific help page, easing navigation.

When an error has been captured, an error report is generated that includes the error message as well as the CoNet app's current settings.

### Network inference workflow

**Figure 1.**
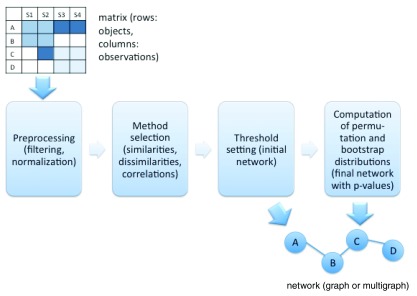
Network inference workflow in CoNet. The network inference workflow in CoNet is divided in preprocessing, initial network computation and assessment of significance.

CoNet takes a presence/absence, count or abundance matrix as input, where rows represent the objects of interest and columns their observations across locations or time points. Optionally, a second input matrix can be provided. This is of interest when two different measurements have been made for the same samples, for instance counts of microorganisms and concentrations of metabolites. CoNet's output consists of a network where significantly associated objects are connected by edges. Figure 1 summarizes the network inference workflow in CoNet.

Depending on the data type, a number of filters needs to be applied. For instance, for 16S rDNA count data, taxa with too few non-zero observations need to be removed and the data needs to be normalized or rarefied to account for sequencing depth differences. In the next step, the user can select from a number of different correlations (Pearson, Spearman, Kendall), similarities (mutual information, Steinhaus, distance correlation etc.) or dissimilarities (Kullback Leibler, Euclidean, Bray Curtis, Jensen-Shannon *etc.*) to score the association strength between the objects. A brief comparison of selected association measures is provided in Table 1. Except for mutual information, these association measures allow assigning a positive or negative sign to a predicted relationship, which reflects whether the abundance distributions of the two objects are significantly more similar or dissimilar than expected at random. In the first case, the relationship is represented by a green edge and in the second by a red edge. For mutual information, which neither quantifies similarity nor dissimilarity, but is a general measure of dependency, the edge is not colored. However, if a mutual information edge is merged with other measure-specific edges connecting the same node pair, the resulting edge will be colored according to these other edges. In general, if measures disagree on the sign, the edge is discarded.

**Table 1.** Comparison of selected association measures for abundance data.

Measure	Value range	Strengths	Weaknesses
Pearson correlation	[-1,1]	More sensitive than other measures. Defined for binary data (Phi coefficient). Defined value range.	Biased by compositionality and matching zeros ( Friedman & Alm, 2012).
Spearman correlation	[-1,1]	Robust to outliers. Defined value range.	Biased by compositionality and matching zeros ( Friedman & Alm, 2012).
Kendall correlation	[-1,1]	Robust to outliers. Defined value range.	Biased by compositionality and matching zeros ( Friedman & Alm, 2012).
Mutual information	[0,INF]	Better able to detect non-linear dependencies than other measures.	Estimation requires large sample number. Estimation debated ( Fernandes & Gloor, 2010). As a general measure of dependency, it does not determine the type of the relationship (negative or positive correlation)
Bray-Curtis dissimilarity	[0,1]	Robust to compositionality and matching zeros. Defined value range.	Sensitive to outliers. Not defined for negative values.
Kullback-Leibler dissimilarity	[0,INF]	Robust to compositionality and matching zeros.	Treatment of zeros necessary to avoid negative infinities. Sensitive to outliers. Not defined for negative values.

For presence/absence (also termed incidence) data, the hypergeometric distribution or Jaccard distance can be chosen for the same purpose. CoNet's special strength is its capability to combine multiple such measures and/or to combine these measures with other network inference algorithms, e.g. those implemented in minet. The idea behind such an ensemble approach to network inference is to exploit the fact that different methods make different mistakes. If erroneous edges predicted by one method are not supported by the others, they can be filtered out, thereby reducing the number of false positives. The thresholds for the measures can be either set manually (using sliding windows for bounded measures) or automatically, by specifying the desired number of edges in the output network. The network can then be displayed either as a multigraph (with as many edges between two objects as selected measures) or as a graph (where scores of individual measures are combined). Optionally, the significance of the associations can be computed, *e.g.* with a permutation test or with the ReBoot method developed in Faust *et al.*, 2012. Multiple testing correction can be performed with either Bonferroni or Benjamini-Hochberg procedures and is only applied to the edges in the initial network. However, the initial edge number can be set sufficiently high or the thresholds sufficiently low that the initial network consists of all possible edges.

CoNet offers various voting systems to combine networks obtained from different measures, including majority voting as well as weighted voting ( Kittler, 1998). Majority voting is implemented in CoNet via the option minsupport. For instance, if four measures were used and minsupport is set to three, an edge will be retained if three out of four measures detected it for the given thresholds and level of significance, corresponding to a majority vote. Alternatively, a more stringent voting can be employed, where an edge is only retained if all measures agree on it (intersection). Weighted voting is implemented in CoNet through the various p-value merging strategies. The Brown p-value merging ( Brown, 1975) is the voting system recommended for CoNet, because it takes the dependency among measures into account. Majority voting assumes independence of measures, which is not true for a number of measures, such as the correlation measures.

### Special features

CoNet offers a series of features that distinguish it from other network inference tools, such as its support for object groups. This feature allows a user to assign objects to different groups ( *e.g*. metabolites and enzymes). Relationships can then be inferred only between different object types (resulting in a bipartite network) or only within the same object type. CoNet's treatment of two input matrices is built upon this feature.

Furthermore, CoNet can handle row metadata, which allows for instance to infer links between objects at different hierarchical levels ( *e.g.* between order Lactobacillales and genus Ureaplasma) while preventing links between different levels of the same hierarchy (e.g. Lactobacillales and Lactobacillaceae). CoNet can also read in sample metadata such as temperature or oxygen concentration. When sample metadata are provided, associations among metadata items and between taxa and metadata items are inferred in addition to the taxon associations. Metadata are then represented as additional nodes in the resulting network. In addition, CoNet recognizes abundance tables generated from biom files ( McDonald *et al.*, 2012) and, in its Cytoscape 3.× version, reads biom files in HDF5 format directly, using the BiomIO Java library ( Ladau). Taxonomic lineages in biom files or biom-derived tables are automatically parsed and displayed as node attributes of the resulting network. For instance, the lineage "k__Bacteria; p__Firmicutes; c__Bacilli; o__Lactobacillales; f__Lactobacillaceae; g__Lactobacillus; s_Lactobacillus acidophilus" of an operating taxonomic unit with identifier 12 would create a kingdom, phylum, class, order, family, genus and species attribute in the node property table for node OTU-12, filled with the corresponding values from the lineage. CoNet also computes a node's total edge number as well as the number of positive and negative edges, the total row sum and the number of samples in which the object was observed (e.g. was different from zero or a missing value).

To ease the selection of suitable preprocessing steps, CoNet can display input matrix properties and recommendations based on them. Importantly, CoNet can also handle missing values, by omitting sample pairs with missing values from the association strength calculation. Finally, CoNet supports a few input and output network formats absent in Cytoscape, including adjacency matrices (import), dot (the format of GraphViz ( http://www.graphviz.org/)) and VisML (VisANT's format ( Hu *et al.*, 2013)) (both for export).

## Results

### Use case: microbial relationships in the arctic soil

We demonstrate the abilities of the CoNet app on a real-world example taken from the Qiita database ( Qiita database). The Qiita database, which merges the previously separated QIIME and EMP databases, is a rich resource for processed 16S rDNA sequence data: each study is accompanied by a microbial count file in biom format computed from the raw sequence data with the QIIME pipeline ( Caporaso *et al.*, 2010).

In our example, we will demonstrate how to build an association network from microbial count data obtained from arctic soil samples ( Chu *et al.*, 2010). This data set was chosen for its sample number (sufficient to compute associations but short run times) as well as for the biological insights that are gained from the network analysis. The example showcases the CoNet app's ability to compute associations between higher taxonomic levels and to take environmental metadata into account, which is important for the interpretation of predicted microbial relationships.

In the Qiita database, the arctic soil study can be found under the title "Soil bacterial diversity in the Arctic is not fundamentally different from that found in other biomes" (study identifier: 104, see Supplementary material). This data set consists of 4,022 operating taxonomic units and 52 soil samples from the arctic tundra, which were sequenced with Roche FLX using primers targeting the V1V2 region of the 16S rDNA. The processed data can be downloaded from the Qiita study page (in Data Types, click on 16S, then click on the URL appearing below, expand the Files network, click on the file object containing BIOM in its name and then download the file with suffix .biom). The study also provides a mapping file with sample metadata (on the Qiita study page, click Sample Information and then the Sample Info button). We extract the pH of each sample by loading the sample information file into Excel, selecting the sample_name and ph columns and saving them to a separate, tab-delimited file.

### Combining multiple measures

The CoNet app is composed of the main window and several menus, including a "Data menu" with input and output options, a "Preprocessing and filter" menu, a "Methods menu" to select network construction methods, a "Merge menu" where the user can specify how results from different network construction methods should be merged, a "Randomization menu" for the assessment of edge significance and finally a "Config menu" for configuration.

In the following, we will build a network from the arctic tundra biom file. First, in the "Data menu", the arctic tundra biom file is selected and the option "Biom file in HDF5" is enabled (direct biom file parsing is only supported in the Cytoscape 3.× version of the CoNet app). In the sub-menu "Metadata and Features", the option "explore links between higher-level taxa" is enabled together with the option "Parent-child exclusion" to compute correlations between higher-level taxa while preventing edges between taxa within the same lineage (e.g. Lactobacillales and Lactobacillaceae). Sample metadata (pH in this case) are passed to the CoNet app via the "Select file" button in the "Features" corner of the "Metadata and Features" sub-menu. Both "Transpose" and "Match samples" need to be enabled to convert sample metadata into rows and to match sample metadata identifiers to biom file identifiers.

In the "Preprocessing and filtering menu", the parameter "row_minocc" is set to 20 to discard taxa with less than 20 non-zero values across samples. The sum of the discarded rows can be kept by enabling "Keep sum of filtered rows". In addition, "col_norm" is activated to divide each matrix entry by the sum of its corresponding column, thus avoiding the inference of spurious links due to sequencing depth differences.

In the "Methods menu", Pearson, Spearman, Bray Curtis, Kullback Leibler and mutual information are selected. Their thresholds can be automatically set such that 1,000 top-scoring and 1,000 bottom-scoring edges (for anti-correlations) are included for each measure in the initial network, by typing "1000" as the value of the edge selection parameter and enabling "Top and bottom" in the "Threshold setting" sub-menu. At this stage, pushing "GO" will result in a multigraph, where microbial taxa are connected by up to five different measure-specific edges.

### Assessment of edge significance

The statistical significance of edgesis computed in two CoNet launches, the first of which generates the permutation distributions and an intermediate network and the second the bootstrap distributions and the final network.

For the first launch, the user selects the "edgeScores" routine in the "Randomization menu", with "shuffle_rows" as resampling parameter, and enables "Renormalize". This last option alters the computation of permutation distributions for correlation measures by introducing a renormalization step that mitigates the compositionality bias ( Faust *et al.*, 2012). The user then specifies a folder and a file name to export permutation scores and enables "Save randomizations" in the "Save" corner of the "Randomization menu". Pushing "GO" will then launch the computation of edge- and measure-specific permutation distributions. Permutation alone is sufficient to set p-values on the edges, but we found that a combination of permutation and bootstrap is more stringent ( Faust *et al.*, 2012). The network generated in this first step should be considered as an intermediate result.

**Figure 2.**
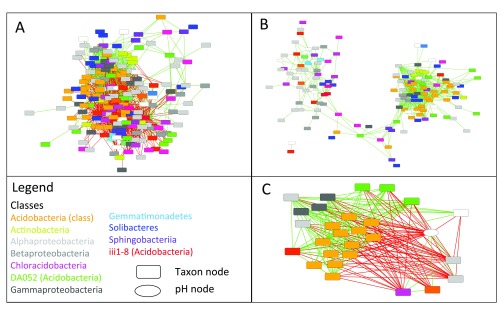
Network inferred with CoNet from the arctic tundra sequencing data set. **A**: Result network obtained for bacterial counts from the arctic soil 16S rDNA example data set, downloaded from the Qiita database. **B**: Same as A, but with negative edges (red) discarded. The remaining positive edges (green) form clusters with different microbial composition. **C**: Neighbors of the pH node form two clusters: one correlated and the other anti-correlated to pH, which reflects the opposite pH preferences of the cluster members. The legend lists the colors of taxonomic classes; taxon nodes in white represent operating taxonomic units above class level.

In order to compute bootstrap distributions and the final network, the user prepares a second CoNet launch, by selecting the "bootstrap" resampling method and a p-value merging method, for instance "brown" ( Brown, 1975), in the "Randomization menu". P-value merging will unite measure-specific p-values for the same edge into a single edge-specific p-value. "Renormalize" is disabled and "benjaminihochberg" is selected as the multiple testing correction method. In the "Save" corner of the "Randomization menu", another file name should be specified to store bootstrap distributions in a separate file. P-values of the final network are computed from both permutation and bootstrap distributions, thus previously generated permutation distributions have to be loaded into the CoNet app. This is done by selecting the permutation file generated in the previous step with the "Load null distributions" button. Pushing "GO" will then result in the final network, shown in Figure 2A.

For this use case, permutation and bootstrap distributions are computed with 100 iterations each. In application cases, we usually increase the iteration number to 1000. However, since the p-value is computed parametrically as the distance between the permutation and the bootstrap distribution, the number of iterations is less critical than for a non-parametric permutation test. According to our previous observations, a network computed with 100 iterations does not differ much from a network computed with 1000 iterations.

The CoNet app does not layout resulting networks, to leave the choice of the (potentially time-consuming) layout algorithm to the user. Here, the "Organic" layout from yFiles was applied and nodes were colored according to their class using Cytoscape's node coloring functionality. The strength of the association, i.e. the merged, multiple-testing-corrected p-value (or q-value), can be visualized as edge width. The continuous mapping function in Cytoscape allows assigning small edge widths to large p-values and large edge widths to small p-values.

Once permutation and bootstrap distributions have been computed, network generation can be quickly repeated by loading both distributions via the "Load null distributions" and "Load randomization file" buttons, respectively. Figure 2B shows the same network re-generated from pre-computed distributions, but with "positive edges only" enabled in the "Preprocessing and filter menu". Figure 2C displays the neighbors of the pH node, which were selected and instantiated as a separate network using Cytoscape's node selection function "First neighbors of selected nodes" for undirected networks.

The computation of permutation and bootstrap distributions took ~5 minutes each for 100 iterations on a standard laptop.

Input and settings files for the use case can be found in the Supplementary material.

## Discussion

### Insights into arctic soil microbiota

After removal of negative edges, the arctic soil network forms two prominent clusters ( Figure 2B), which are enriched with representatives of different classes, such that one cluster features mostly members of the Solibacteres and Acidobacteria, whereas the other consists mostly of Alphaproteobacteria and Chloracidobacteria. When examining the neighbors of the pH node ( Figure 2C), members of the former cluster are found to be anti-correlated to pH, whereas members of the latter are correlated to it. Thus, network analysis helps to identify pH as a major driving factor for microbial soil communities, as has been found previously ( Fierer & Jackson, 2006). The correlations with pH have also been described by the authors of the soil study ( Chu *et al.*, 2010). However, network analysis adds more details (correlations are computed on lower taxonomic levels) and discovers additional taxonomic groups impacted by pH, e.g. Chloracidobacteria. Furthermore, network inference suggests candidates for cross-feeding. For instance, the neighboring nodes of *Bradyrhizobium*, a nitrogen fixer that produces ammonium, may represent taxa that depend on ammonium as main nitrogen source.

### Beyond arctic soil

Previously, we studied the microbial community structure in the human gut ( Human Microbiome Project Consortium, 2012) and the open ocean ( Lima-Mendez* *et al.*, 2015) with CoNet. In both cases, we summarized nodes into higher-level units that were connected when a significant number of their members was inter-linked. In this way, we could group body sites into microbial habitats, identify hub classes in the oral cavity and highlight the importance of competitive and parasitic interactions in plankton communities. We also applied CoNet to build time-varying networks ( Faust *et al.*, 2015b) and to compare networks from different environments ( Faust *et al.*, 2015a). Other authors used the CoNet app to investigate the structure of microbial communities on coral surfaces ( Meyer *et al.*, 2014) or in lakes ( İnceoğlu *et al.*, 2015). In summary, the CoNet app is a versatile tool that is widely applied to derive ecological hypotheses from sequencing data.

### Related apps

The CoNet app offers mostly similarity-based network inference. Complementary apps that implement various Bayesian network inference algorithms are Cyni Toolbox ( http://www.proteomics.fr/Sysbio/CyniProject), bayelviraApp ( http://apps.cytoscape.org/apps/bayelviraapp) and MONET ( Lee & Lee, 2005). ARACNE ( http://apps.cytoscape.org/apps/aracne) exploits mutual information to build networks ( Margolin *et al.*, 2006). ExpressionCorrelation ( http://www.baderlab.org/Software/ExpressionCorrelation) and MetaNetter ( http://apps.cytoscape.org/apps/metanetter) also offer similarity-based network inference techniques, in case of the former specialized to gene expression and in the latter to metabolomics data. Results from these different network inference approaches could be combined with Cytoscape tools such as Merge Networks.

## Conclusion

In this article, we have demonstrated the CoNet app on a typical 16S data set. Alternative use cases are for instance the inference of function networks ( *i.e.* co-occurrence of orthologous gene groups) from metagenomics or metatranscriptomics data or taxon-metabolite networks from 16S and metabolomics data.

We hope that CoNet's integration into Cytoscape will lower the barrier for its employment by users less familiar with the command line version. Due to its flexibility and comprehensiveness, CoNet can be useful in a variety of applications and we thus hope it will find a broad user base.

## Software availability

CoNet app page: http://apps.cytoscape.org/apps/conet

CoNet tool web page: http://systemsbiology.vub.ac.be/conet

Latest source code: http://sourceforge.net/projects/conet/

Archived source code as at the time of publication: Zenodo, Biological network inference in Cytoscape, doi: 10.5281/zenodo.55715 ( Faust & Raes, 2016)

License: GNU General Public License version 2.0

## Supplementary material

Use case data in CoNet app: inference of biological association networks using Cytoscape.

This file contains microbial count data, sample metadata, permutation settings and bootstrap settings associated with this submission. Description of each dataset is provided in the text file.
