# Supplementary material for: CoNet app: inference of biological association networks using Cytoscape
Source: F1000Res. 2016 Oct 14;5:1519. Originally published 2016 Jun 27. [Version 2] doi: 10.12688/f1000research.9050.2 (PMC5089131; doi:10.12688/f1000research.9050.2)
Supplement: Peer review history

# Peer review: Referee response for version 2

**DOI:** 10.5256/f1000research.10536.r17150

**Published:** 2016-10-24

**Authors:** Alexander Eiler

**Affiliations:** 1 Department of Ecology and Genetics, Uppsala University, Uppsala, Sweden

I do not have any further comments - the authors made a great effort to detail the different statistical inferences and how they can be combined which was the major criticism on the first version.

I have read this submission. I believe that I have an appropriate level of expertise to confirm that it is of an acceptable scientific standard.

# Peer review: Referee response for version 2

**DOI:** 10.5256/f1000research.10536.r16998

**Published:** 2016-10-17

**Authors:** Paul Wilmes, Anna Heintz-Buschart

**Affiliations:** 1 Luxembourg Centre for Systems Biomedicine (LCSB), University of Luxembourg, Luxembourg City, Luxembourg

The authors have addressed all previously raised issues.

We have read this submission. We believe that we have an appropriate level of expertise to confirm that it is of an acceptable scientific standard.

# Peer review: Referee response for version 1

**DOI:** 10.5256/f1000research.9740.r15254

**Published:** 2016-09-12

**Authors:** Alexander Eiler

**Affiliations:** 1 Department of Ecology and Genetics, Uppsala University, Uppsala, Sweden

Be more precious, here. "The idea behind such an ensemble approach to network inference is to exploit the fact that different methods make different mistakes." 

 These are different statistical inferences so based on the underlaying algorithms results will be different. Same may be better suited for parametric or none-parametric data, some perform better with larger or smaller sample numbers. The different methods have also different statistical power do identify significances. Some may produce more false positives or false negatives than others. Some guidance and references to statistical literature could be provided in the article.

 I really liked to see an implementation that calculates false discovery rate (after Benjamin Hochberg) over all statistical comparisons.

I have read this submission. I believe that I have an appropriate level of expertise to confirm that it is of an acceptable scientific standard, however I have significant reservations, as outlined above.

# Response

**Published:** 2016-10-07

**Authors:** Karoline Faust

**Affiliations:** VUB, Belgium

"These are different statistical inferences so based on the underlaying algorithms results will be different."

 Thanks for pointing this out. We have now added an overview table of the strengths and weaknesses of selected measures available in CoNet. We also added a paragraph that discusses the different ways in which these measures can be combined in CoNet.

 "I really liked to see an implementation that calculates false discovery rate (after Benjamin Hochberg) over all statistical comparisons."

 CoNet does allow computing false discovery rate over all statistical comparisons either by setting the number of initial edges sufficiently high or by setting the thresholds on the individual measures sufficiently low. We have added this remark to the article.

 Despite the fact that multiple testing correction is in most cases only applied after edges are discarded through initial filtering, CoNet is among the microbial network inference tools with the lowest false positive rates tested in Weiss et al., The ISME Journal 2016 ( https://www.ncbi.nlm.nih.gov/pubmed/26905627, supplementary Figure 10) .

# Peer review: Referee response for version 1

**DOI:** 10.5256/f1000research.9740.r14620

**Published:** 2016-07-13

**Authors:** Paul Wilmes, Anna Heintz-Buschart

**Affiliations:** 1 Luxembourg Centre for Systems Biomedicine (LCSB), University of Luxembourg, Luxembourg City, Luxembourg

The article describes a Cytoscape plugin “CoNet app” designed for the inference of networks from microbial abundance or incidence matrices. The effort combining a versatile network inference tool with a user-friendly and widely used network visualization and analysis framework, such as Cytoscape is very valuable to the community.

 I would suggest certain improvements to the article to make it in itself more valuable for potential users to judge the applicability of the plugin to their datasets.   

**Introduction:** As the authors of the plugin are well aware (being co-authors of “Correlation detection strategies in microbial data sets vary widely in sensitivity and precision” ^1^), co-abundance or co-occurrence analysis is an approach to ecological data interpretation that is not without caveats and as such, the article is lacking both mention of limitations of the approach and references to the successful use cases of earlier versions of CoNet. I suggest including both in the introduction.

 The introduction also does little to explain the approach to potential users who are not familiar with the concept. E.g. the sentence “More specifically, co-occurrence analysis detects significant co-occurrences or mutual exclusions across samples, which are interpreted as representing ecological relationships such as mutualism or competition or being due to similar responses to environmental factors.” mixes up observations and analyses with interpretation. Similarly, relating to the first sentence of the introduction, microbial count data are not obtained from relative abundances, but microbial counts taken to infer relative abundances (the sentence is also ambiguous as to what these abundances are relative to). Furthermore, the second but last sentence of the introduction “The large number of microbial count tables resulting from the multitude of recent sequencing projects…” can be interpreted to advise for the co-analysis of results from different studies, which is most often not possible. These parts should be revised for clarity.

**Methods/Implementation:** More details on the algorithms would be useful, or alternatively references to other publications which describe CoNet, as relates to the following points:

- “its capability to combine multiple such measures and/or to combine these measures with other network inference algorithms”,
- “CoNet can also parse sample metadata such as temperature or oxygen concentration, which are then correlated with the objects in the input matrix while being excluded from normalization.” and
- “Phylogenetic lineages in these tables are automatically parsed”. Also, what are positive and negative edges? How is mutual information integrated with measures which can be positive or negative?

**Use case**: It would be helpful to shortly describe the size of the dataset (number of OTUs and number of samples) as part of the sentence “This data set was chosen for its sample number (sufficient to compute associations but short run times) as well as for the biological insights that are gained from the network analysis.” A general advice on the required sample number and or relationship between numbers of analyzed features and sample numbers would also be helpful. In addition, are the 100 iterations performed in this example a realistic number of iterations to be used in such an analysis?

 The formulation “The significance of edges, that is their p-values” is a bit unfortunate. On a similar note, next to the permutations, is there a way in CoNet or the CoNet app to assess association strengths? An example of how the assessment of edge significance affects network size and structure would be informative.

 Figures: The large heading in Figure 1 should be removed. Figure 2 would benefit from a heading. The labels of figure 2 are not legible. It is unclear from the text and not mentioned in the legend, how the “classes” used for coloring nodes are defined. Are these classes in the taxonomic sense or different kinds of data? The color scheme for positive and negative edges should be explained. In panel C, the pH node should be more clearly pointed out.

**Small comments:**

 The referenced “Brown 1975” does not appear in the references.

 The capitalization of “P-value” is inconsistent.

 As the buttons in the app are actually called that, refer to “Data menu”, “Preprocessing and filter menu” etc.

We have read this submission. We believe that we have an appropriate level of expertise to confirm that it is of an acceptable scientific standard, however we have significant reservations, as outlined above.

## References

[1] WeissSVan TreurenWLozuponeCFaustKFriedmanJDengYXiaLCXuZZUrsellLAlmEJBirminghamACramJAFuhrmanJARaesJSunFZhouJKnightR: Correlation detection strategies in microbial data sets vary widely in sensitivity and precision. *ISME J*.2016;10(7) : 10.1038/ismej.2015.235 1669-81 10.1038/ismej.2015.235 26905627PMC4918442

# Response

**Published:** 2016-10-07

**Authors:** Karoline Faust

**Affiliations:** VUB, Belgium

"The effort combining a versatile network inference tool with a user-friendly and widely used network visualization and analysis framework, such as Cytoscape is very valuable to the community."

 We would like to thank the reviewer for this appreciation of our work.

**Introduction**

 In response to the reviewers' comments, we have rewritten the introduction, thereby rephrasing problematic sentences, pointing out limitations of microbial network inference and citing the evaluation. We also added a paragraph in the discussion to mention applications of CoNet.

**Methods/Implementation**

- “its capability to combine multiple such measures and/or to combine these measures with other network inference algorithms”,

We included an overview table comparing selected measures of association. We also added a paragraph on how measures can be combined in CoNet.

- “CoNet can also parse sample metadata such as temperature or oxygen concentration, which are then correlated with the objects in the input matrix while being excluded from normalization.”

We improved this explanation of CoNet's treatment of sample metadata.

- “Phylogenetic lineages in these tables are automatically parsed”.

We provided an example to better explain what we mean.

- Also, what are positive and negative edges? How is mutual information integrated with measures which can be positive or negative?

We added an explanation.

**Use case**

 The OTU number was added to the following sentence (which already listed the sample number):

*This data set consists of 4,022 operating taxonomic units and 52 soil samples from the arctic tundra, which were sequenced with Roche FLX using primers targeting the V1V2 region of the 16S rDNA.*

 "A general advice on the required sample number and or relationship between numbers of analyzed features and sample numbers would also be helpful."

 In general, the number of false positives increases with decreasing sample number. While assessment of significance counter-balances this effect, it is unreasonable to compute a correlation from a few observations only, even if it is strongly significant. However, we cannot provide a formula to compute where exactly to put the cut-off.

 "In addition, are the 100 iterations performed in this example a realistic number of iterations to be used in such an analysis?"

 We saw previously that there is no big difference between networks computed with 100 or 1000 iterations. The reason is that we are not computing p-values from a pure permutation test, where small p-values can only be reached by performing a sufficient number of iterations. Instead, we compute the p-value parametrically as the mean of the permutation distribution under the bootstrap distribution. Estimating the mean and standard deviation of normal distributions is less sensitive to iteration number than computing parameter-free p-values. We added this explanation to the text.

 "The formulation “The significance of edges, that is their p-values” is a bit unfortunate. On a similar note, next to the permutations, is there a way in CoNet or the CoNet app to assess association strengths? An example of how the assessment of edge significance affects network size and structure would be informative."

 The p-value is an assessment of association strength. So are the scores of the measures themselves, e.g. Pearson's r and Spearman's rho, which are correlated with the p-value. We have added a remark explaining this to the text.

 Assessing the significance usually discards edges from the initial network, in some cases even removing all initial edges. The number of edges removed depends on the initially selected thresholds. In the use case, the initial network consists of 10000 edges, 1546 of which remain after assessment of significance and merging of measure-specific p-values into a single p-value. The exact edge number in the final network may vary slightly from run to run, due to variations in the permutation and bootstrap distributions.

 "Figures: The large heading in Figure 1 should be removed."

 This heading was not intended as a Figure heading but as a heading to divide the text. We improved the layout and added headers to Figure 1 and 2.

 "Figure 2 would benefit from a heading. The labels of figure 2 are not legible."

 Our aim here was to show the networks as obtained with the CoNet app when executing the use case, but we understand the point of the reviewer. As a compromise, we have now removed the labels and added a class-level color code.

 "It is unclear from the text and not mentioned in the legend, how the “classes” used for coloring nodes are defined. Are these classes in the taxonomic sense or different kinds of data?"

 These are taxonomic classes. We clarified this in the caption of Figure 2.

 "The color scheme for positive and negative edges should be explained."

 We added an explanation of the color scheme in the main text and to the caption of Figure 2.

 "In panel C, the pH node should be more clearly pointed out."

 The pH node stands out by differing in shape from the taxon nodes. We have clarified this by adding a legend to Figure 2.

**Small comments:**

 "The referenced “Brown 1975” does not appear in the references."

 We excuse for this oversight. We have added the reference.

 "The capitalization of “P-value” is inconsistent."

 We now use p-value with a lower case p, unless it is the first word of a new sentence, where we use the upper case P.

 "As the buttons in the app are actually called that, refer to “Data menu”, “Preprocessing and filter menu” etc. "

 Done
